# Deep Dive Into the Effects of Food Processing on Limiting Starch Digestibility and Lowering the Glycemic Response

**DOI:** 10.3390/nu13020381

**Published:** 2021-01-26

**Authors:** Gautier Cesbron-Lavau, Aurélie Goux, Fiona Atkinson, Alexandra Meynier, Sophie Vinoy

**Affiliations:** 1 Nutrition Research, Mondelēz International R&D, 91400 Saclay, France; gautiercesbron@hotmail.com (G.C.-L.); aurelie.goux@mdlz.com (A.G.); alexandra.meynier@mdlz.com (A.M.); 2 School of Life and Environmental Sciences and the Charles Perkins Centre, University of Sydney, Sydney, NSW 2006, Australia; fiona.atkinson@sydney.edu.au

**Keywords:** food process, glycemic response, digestibility, starch, micro imaging, X-ray diffraction

## Abstract

During processing of cereal-based food products, starch undergoes dramatic changes. The objective of this work was to evaluate the impact of food processing on the starch digestibility profile of cereal-based foods using advanced imaging techniques, and to determine the effect of preserving starch in its native, slowly digestible form on its in vivo metabolic fate. Four different food products using different processing technologies were evaluated: extruded products, rusks, soft-baked cakes, and rotary-molded biscuits. Imaging techniques (X-ray diffraction, micro-X-ray microtomography, and electronic microscopy) were used to investigate changes in slowly digestible starch (SDS) structure that occurred during these different food processing technologies. For in vivo evaluation, International Standards for glycemic index (GI) methodology were applied on 12 healthy subjects. Rotary molding preserved starch in its intact form and resulted in the highest SDS content (28 g/100 g) and a significantly lower glycemic and insulinemic response, while the three other technologies resulted in SDS contents below 3 g/100 g. These low SDS values were due to greater disruption of the starch structure, which translated to a shift from a crystalline structure to an amorphous one. Modulation of postprandial glycemia, through starch digestibility modulation, is a meaningful target for the prevention of metabolic diseases.

## 1. Introduction

There has been increasing attention on food processing methods, as transforming food products can modify the physiological fate of their components. There is growing controversy regarding the potential risks of some food processing technologies on health [1,2]. For several decades, since the food processing industry began to play a significant role in food production, processed foods have formed a significant part of our daily diets [3].

Starch is a major daily source of carbohydrates, and represents approximately 50–70% of human energy intake. Food starch is derived from cereals (e.g., wheat, maize, rice, barley, and buckwheat), tubers (e.g., potatoes and cassava) and legumes (e.g., peas, lentils, kidney beans, and mung beans). It consists of a semi-crystalline material produced by plants that forms roughly spherical granules in plant tissues [4]. The sizes and shapes of the granules depend on the botanical origin, as well as the relative amounts of amylose (a straight chain polymer composed of α-1,4-linked glucose units) and amylopectin (a branched chain polymer that contains α-1,6-linked glucose units at the branch point and α-1,4 links in the linear regions) [5,6]. The digestibility of starch in its natural state varies greatly depending on these variables [7,8,9].

The food processing technologies and cooking methods applied to cereal products result in these products exhibiting a wide range of available starch digestibility profiles [10,11,12,13]. During food manufacturing, heat, dough moisture, and pressure can dramatically modify the digestibility of starch in processed foods [14,15]. The rate and extent of starch digestion are mostly measured in vitro using a method developed by Englyst et al. [16,17] that classifies starch into three major fractions: rapidly digestible starch (RDS), slowly digestible starch (SDS), and resistant starch (RS). During food processing, starch granules undergo dramatic changes when heated in the presence of high moisture [18,19]. As the temperature increases, hydrogen bonds between the starch chains are disrupted, and water is absorbed by the starch granule. This leads to swelling of the granule, which is followed by amylose leaching. Starch dissolves progressively, gradually increasing the viscosity of the solution. Gelatinization leads to the formation of a starch paste [20]. There is an inverse relationship between SDS and the gelatinization of starch in cereal products [21]: cereal products with the highest levels of SDS have the lowest degree of starch gelatinization. Thus, controlling food-processing conditions can prevent SDS loss by limiting the extent of starch gelatinization. The combination of high moisture levels and high temperatures (e.g., during baking) or high pressure and shearing (e.g., during extrusion) converts nearly all SDS to RDS [22]. The addition of fiber or bran to cereal matrices often requires the addition of more water to the dough, which may promote starch gelatinization, thereby facilitating the conversion of SDS to RDS during food processing [23].

Complementary approaches can be used to observe structural modifications to starch during food processing. These effects can be observed and measured by X-ray diffraction, micro-X-ray-tomography, and scanning electron microscopy [24,25,26]. Such methods are often used to study changes in starch crystallinity without exploring the effects of these changes in vivo [27,28]. Indeed, most studies to date have focused on a specific type of starch or a specific processing technology; however, very few have performed an extensive analysis of the effects of different processing technologies on starch structure and digestion kinetics.

Furthermore, postprandial glycemic excursions, as well as the related phenomena of insulinemia and lipemia, have been implicated in the etiology of chronic metabolic diseases such as type 2 diabetes [29,30]. In addition, several studies have demonstrated the beneficial effect that adding fiber to cereal products has on the glycemic response [23,31] and long-term health [32,33].

At a mechanistic level, comprehensive studies have described the potentially deleterious effects of increased carbohydrate intake on the glycemic response [34,35,36]. Thus, the postprandial impact of starchy foods should be investigated in greater detail. Few human studies comparing the physiological effects of starch-based products with different SDS contents have demonstrated a strong link between in vitro starch digestibility and postprandial plasma glucose and insulin responses [37,38,39,40].

The innovative goal of this work was to determine the relationships between complementary parameters involved in starch digestibility, from its observable structure inside food products to its in vivo metabolic fate, to the physiological response to consumption of cereal-based food products. The findings from this study will improve our understanding of the impact of food processing on human health, which is currently a topic of considerable debate.

## 2. Materials and Methods

### 2.1. Test Products

Seven wheat flour–based products were prepared using four different technologies. “Technology” is used here to refer to the series of processes involved in making a finished product. These processes include the ingredients used, the mixing and cooking conditions, and the machines used to make the products. The resulting products differed greatly in aspect, taste, and texture. Four technologies were tested with and without wheat bran to evaluate the impact of bran on starch digestibility: two rotary-molded biscuits, with and without wheat bran (RB and RnB, respectively); two extruded products, with and without wheat bran (EB and EnB, respectively); two soft-baked cakes, with and without wheat bran (SB and SnB, respectively); and a rusk-type product without wheat bran (Rk) only. The rotary-molded products, soft-baked cakes, and rusk were made at the Mondelēz International R&D center in Saclay, France. The extruded products were made in collaboration with Welience, based at the University of Bourgogne, Dijon, France.

All products were made using the same ingredients in similar quantities, although some adjustments were made to accommodate the specific requirements of certain technologies. Eggs and glycerin were added to the soft-baked cake recipes (approximately 13% and 6%, respectively). Different amounts of water were added to the dough as needed (from 0% to 12%). Finally, 6% of yeast was added to the rusk recipe (Table 1).

### 2.2. Nutritional Composition and Starch Digestibility

The nutritional parameters were measured analytically (fiber: AOAC 985-29; total starch: enzymatic method as described in the French standard V18-121; fat and moisture: methods described in the French Decree of 8 September 1977; protein: Kjeldahl method). In vitro starch digestibility was assessed using the SDS method developed by Englyst et al. [17]. This method involves several steps that simulate enzymatic digestion of carbohydrates in the stomach and the small intestine, and measures the release of glucose at several time points. This method made it possible to measure the amounts of different starch and sugar fractions according to their digestibility [16].

### 2.3. Micro-Imaging

Three different micro-imaging methods were carried out by Novitom (Paris, France), a company that specializes in microscopy analysis.

#### 2.3.1. X-ray Diffraction

X-ray diffraction enables analysis of molecular structures at the micrometer scale [41,42]. Samples were partially crushed by hand and introduced into a 2-mm capillary tube. Data were collected using a laboratory diffractometer operated at a voltage of 40 keV and an intensity of 40 mA. The resulting diffraction patterns were then analyzed using FIT2D software and integrated in 360° to obtain a diffraction profile. In addition to the seven samples, the raw flour used in the products was analyzed for comparison.

#### 2.3.2. Micro-X-ray-Tomography

The four products without bran (RnB, EnB, SnB, and Rk) were analyzed by micro-X-ray tomography. Each sample was crushed prior to analysis. Measurements were taken by synchrotron radiation (at the European Synchrotron Radiation Facility, F-38, Grenoble, France), in which the density of a material is reflected by different degrees of X-ray absorption. The resulting images are shown in different shades of gray: denser materials are shown in lighter gray, whereas air bubbles show up as black or very dark gray patches.

#### 2.3.3. Low-Voltage Scanning Electron Microscopy

The electronic microscope was used in Field Emission Gun mode, a technique which requires no prior sample preparation (e.g., coating or crushing). The microscope used was a Zeiss Supra55VP at the Physics Laboratory of Solids (Orsay, France). The imaging was performed with a voltage of 1.8 keV at magnifications of ×200 and ×600.

### 2.4. Human Subjects and the In Vivo Study

Twelve healthy, non-smoking volunteers between 18 and 45 years old participated in a randomized, open trial with a cross over study design at the Human Nutrition Unit within the School of Life and Environmental Sciences at the University of Sydney, Australia. The ethical committee approval number was 2015/082. They consumed test portions of the cereal products containing 50 g of glycemic carbohydrates with 250 mL of Evian water over a 10–12-min period. Capillary blood samples were collected at the time of consumption (T0) and 15, 30, 45, 60, 90, and 120 min later to construct glycemic and insulinemic response curves for each test product. As recommended by the International Standards [43], the subjects selected for the Glycemic Index (GI) test were healthy human subjects of both sex who satisfied two additional inclusion criteria: fasting blood glucose level < 6.0 mM and 2-h blood glucose level following the ingestion of a 50-g glucose solution < 8.9 mM. The mean age and BMI of the study subjects were 27.8 (SD 7.6) years and 21.7 (SD 2.0) kg/m^2^, respectively. The mean fasting blood glucose level and blood glucose level 2 h after ingestion of a 50-g glucose solution were 5.12 (SD 0.25) and 4.60 (SD 0.59) mM, respectively.

Each volunteer consumed one portion of each test product, as well as three servings of a glucose solution (reference food), which corresponded to 10 test sessions in total, separated by a minimum of 1 day of wash out. Detailed descriptions of the portion sizes and macronutrient contents of the seven cereal products and the reference glucose solution are shown in Table 2.

The GI of a given food reflects how much its digestible carbohydrate content raises blood glucose levels. It is defined as the incremental area under the blood glucose response curve (iAUCg) after consumption of a portion of test food providing 50 g of available carbohydrates, and is expressed as a percentage of the average iAUCg for the same amount of carbohydrates from a reference food (glucose) ingested by the same subject on a separate occasion (iAUCg test food/average iAUCg reference food × 100). The iAUCg is the incremental area under the blood glucose response curve (calculated over a 2-h period following ingestion of the test product: 0–120 min), ignoring the area beneath the fasting concentration (as recommended by the International Standard [43]). Other parameters defining the shape of the response curves over 2 h were also investigated, including baseline glycemia and maximum concentration (Cmax(g) of the glycemic response). Moreover, the Insulin Index (II) was calculated (similar to the GI) by measuring the extent to which a food product raised the plasma insulin concentration [44]. The same parameters used to describe the glycemic response curve (incremental area under the blood insulin response curve (iAUC(ins)), baseline insulin, and maximum concentration (Cmax(ins)) were used to describe the insulinemic response.

### 2.5. Statistical Analysis

Descriptive statistics (mean, median, SD, SEM) of all measured and calculated (GI and II) variables related to each food were determined using JMP^®^ Statistics software (version 14). Repeated-measures analysis of variance (ANOVA) was used to determine whether there were any significant differences between the mean GI and II values of the test foods. If a statistically significant product-effect was found, a post hoc multiple comparisons test was performed in order to identify the specific significant differences. For normally distributed data, the Least Significant Difference (LSD) test was used as the post hoc test for multiple comparisons. For non-normally distributed data, a global comparison was performed using the non-parametric Friedman test, taking repeated measures into account.

## 3. Results

### 3.1. Macronutrient Content

For a portion size containing 50 g of available glucose, the protein and fat content of all products was similar, ranging from 4–6 g/portion and 10–12 g/portion, respectively (Table 2). The sugar content was also relatively similar in all food forms (ranging from 14–16 g/portion), except for the soft-baked cakes (SB and SnB), which contained 17 g and 20 g/portion, respectively. The starch content ranged from 28 to 33 g/portion, with the lowest values being found in the soft-baked cakes (SB = 29 g and SnB = 28 g). The fiber content was similar in the products containing bran (RB, EB, and SB) at 4 g/portion, and in the products without bran (RnB, EnB, Snb, and Rk) at 1–2 g/portion.

### 3.2. Starch Fraction Analyses

The starch digestibility analysis revealed major differences between the fractions of starch in the products. The conversion from SDS to RDS occurred to different extents depending on the different food processing techniques. The SDS fraction was much higher, and the RDS was correspondingly much lower in RB and RnB compared to the three other technologies, soft-baked cakes, rusk, and extruded product (Table 3). The resistant starch fractions were similar in all seven products.

### 3.3. Micro-Imaging

#### 3.3.1. X-ray Diffraction

**Figure 1:**
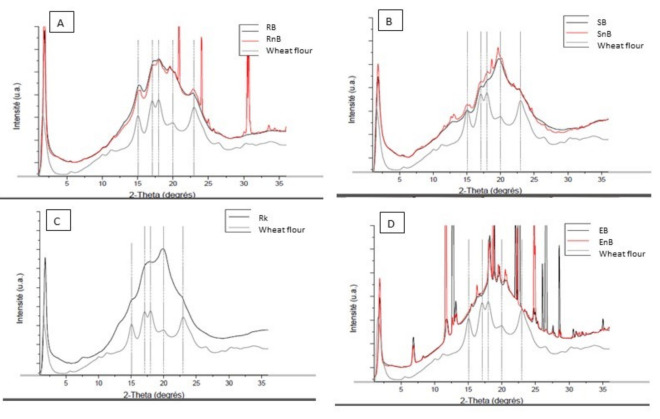
Diffraction pattern of seven wheat flour–based products and raw wheat flour analyzed by X-ray diffraction. (**A**) rotary-molded biscuits, with and without wheat bran (RB and RnB, respectively), and wheat flour; (**B**) soft-baked cakes, with and without wheat bran (SB and SnB, respectively), and wheat flour; (**C**) rusk without bran (Rk), and wheat flour; (**D**) extruded products, with and without wheat bran (EB and EnB, respectively), and wheat flour.

The diffraction pattern of the wheat flour, which was used as a control, displayed a typical A-Type crystallinity profile with peaks at 2-Theta values of 15°, 17°, 18°, and 20°. As shown in graph A in Figure 1, the diffraction patterns of RB and RnB showed the same diffraction peaks as wheat flour, but with different intensities. The RB and RnB patterns overlapped, with an intensity difference of 1.85%, and thus can be considered similar. As shown in graph B, the diffraction patterns of SB and SnB displayed the same peaks that characterize an A-type starch. However, the relative intensity of the starch peaks varied from the previous samples. The peak at a 2-Theta value of 19.5° increased in intensity. Once again, no major structural or crystallinity differences were observed between SB and SnB. The diffraction pattern for Rk, as shown in graph C, followed the same pattern as the previous samples, but the peaks were larger and less intense. A comparable increase in intensity at a 2-Theta value of 19.5° was observed for Rk, SB, and SnB.

As shown in graph D, the diffraction patterns for EB and EnB were very similar to one another, but did not match any of the previous patterns. They were characterized by a multitude of punctual peaks that did not match any commonly known starch diffraction patterns.

#### 3.3.2. Micro-Tomography

**Figure 2:**
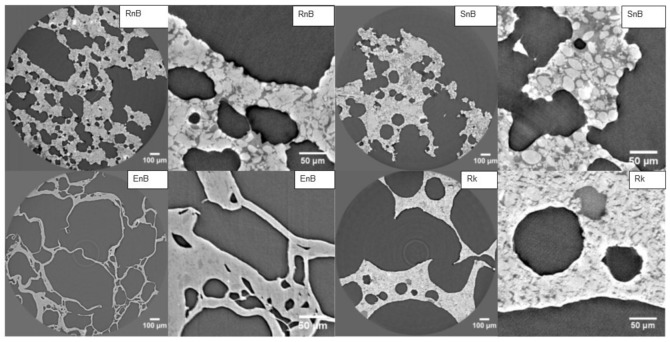
Images obtained by X ray microtomography for the four food technologies tested. Only products without bran were analyzed here: (RnB) rotary-molded biscuit; (EnB) extruded product; (SnB) soft-baked cake; (Rk) rusk. Each product was imaged at two different magnifications (the scale bars are 50 and 100 micrometers).

As shown in Figure 2, the rotary-molded product without bran (RnB) exhibited several interconnected porosities of different shapes and sizes. Intact starch granules were visible inside a less dense matrix. There were also compact or uniform domains that had the same density as starch. Additionally, some very dense spots were observed, which could be individual sugar crystals. The extruded sample (EnB) was very porous, as shown by the large gray patches (Figure 2). The matrix formed continuous walls of varying thickness in which no starch grains could be seen. However, the matrix exhibited two different densities, as indicated by the slightly darker spots within the walls. The soft-baked cake (SnB) resembled the rotary-molded biscuit, with some noticeable differences. The porosities were more rounded or globular in shape, there were fewer well-formed starch granules, and the matrix that these granules were embedded in was less homogenous, as indicated by the wider variety of shades of gray. The rusk (Rk) contained larger porosities than the extruded product, and some of these porosities were filled with less dense material. The matrix seemed to be partly homogeneous, with starch granules that were still distinguishable but appeared deformed or flattened. The matrix also contained a material that was similar in density to the starch.

#### 3.3.3. Electronic Microscopy

**Figure 3:**
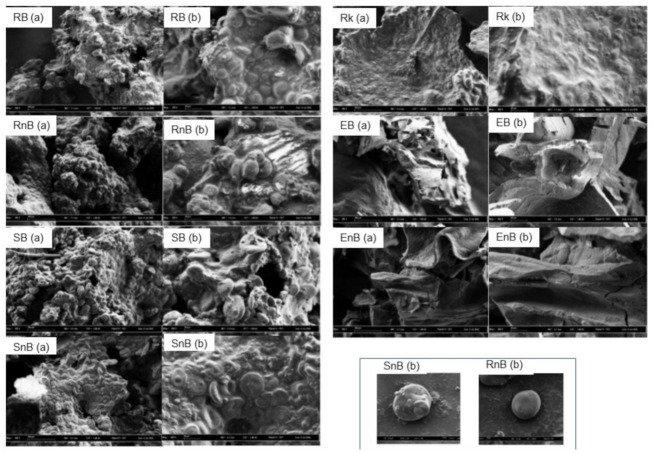
Electronic microscopy images were taken at ×200 (**a**) and ×600 (**b**) magnification for the seven products: rotary-molded biscuits, with and without wheat bran (RB and RnB, respectively); extruded products, with and without wheat bran (EB and EnB, respectively); soft-baked cakes, with and without wheat bran (SB and SnB, respectively), and rusk without wheat bran (Rk). The bottom right box shows starch granules from soft-baked cake without wheat bran (SnB) and rotary-molded biscuit without wheat bran (RB) at ×600 magnification.

In both rotary-molded biscuits (Rb and RnB), starch granules were embedded in a rough matrix but remained visibly intact (Figure 3). The starch granules were oval in shape with a smooth surface overall. Both products were very similar in appearance. In both soft-baked cake products (SB and SnB), two different sizes of starch granules were present in a rough matrix. The interaction between the matrix and the granules seemed fragile, as individual starch granules were also visible. The granules differed from those observed in the rotary-molded products (RB and RnB), as they appeared to be flat, and a minor depression could be seen in the center. The rusk was made up of granules that were completely embedded in a smooth and seemingly homogeneous matrix. The granules, while flattened and sunken in the middle, remained distinguishable from the matrix. The extruded products (EB and EnB) were completely different in appearance from the other products: no starch granules were visible and the matrix seemed to be made up of relatively smooth layers (Figure 3).

### 3.4. Glycemic and Insulinemic Responses

**Figure 4:**
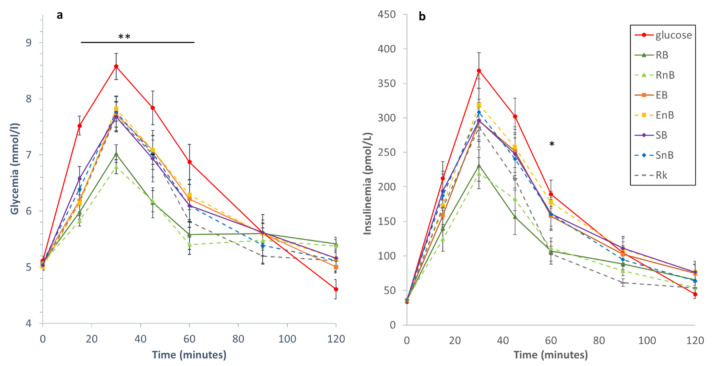
Kinetics of the postprandial (**a**) glycemic response and (**b**) insulin response to the seven cereal-based food products (rotary-molded biscuits, with and without wheat bran (RB and RnB, respectively); extruded products, with and without wheat bran (EB and EnB, respectively); soft-baked cakes, with and without wheat bran (SB and SnB, respectively); and rusk without wheat bran (Rk)) and the glucose solution. *n* = 12 subjects, results are shown as mean ± SEM. Overall analysis of the glycemic and insulin responses kinetics showed a significant difference among products (*p* < 0.001). * *p* < 0.05; ** *p* < 0.01.

The mean kinetics of the 12 subjects’ glucose and insulin concentrations for the seven food products and the reference food are shown in Figure 4. Baseline values for the glycemic and insulinemic responses were not significantly different between the products (Table 4). Overall analysis of the glycemic response kinetics showed a significant difference among products (*p* < 0.001), especially between T15 and T60 (Figure 4a). Mainly, both rotary-molded biscuits (RB and RnB) showed the lowest glycemic responses, and there was no significant difference among the glycemic responses to the soft-baked cakes (SB and SnB), the extruded products (EB and EnB), and the rusk (Rk). Ingestion of the glucose solution led to the highest glycemic curve, as expected. Complementary analysis showed that the lowest Cmax(g) and iAUC(g) were obtained for RB and RnB, while the highest were obtained for EnB and SB (Table 4). Overall analysis indicated that there was a significant difference among the food products’ mean GI values (*p* < 0.0001). Both rotary-molded biscuits (with or without bran) exhibited the lowest GI values, at 47 and 43, respectively, which were significantly lower than those for all the other products (*p* ≤ 0.021 and *p* ≤ 0.004, respectively) (Table 4). The soft-baked cakes with bran and without bran, the rusk, and the extruded product with bran had similar intermediate values of 63, 66, 61, and 66, respectively. The GI value for EnB was significantly higher than those for SB (*p* = 0.026) and Rk (*p* = 0.013), at 77 (Table 4).

Overall analysis of the insulin response kinetics showed that there was a significant difference between the products during the 2-h postprandial period (*p* < 0.001) (Figure 4b). Ingestion of the glucose solution led to a significantly higher insulin response curve compared to the other seven products. The most significant differences among the technologies were observed at T60, when the lowest insulin values were obtained with RB and RnB, compared to the other products. Complementary analysis showed that the lowest Cmax(ins) and iAUC(ins) were obtained with RB and RnB, while the highest were obtained with EnB and SB (Table 4). There was a significant difference among the food products’ mean II values (*p* < 0.0001). The average II values for the rotary-molded biscuits (RB and RnB) were significantly lower than the II values for four of the other products (SB, SnB, EB, EnB; *p* ≤ 0.001). The average II value for Rk was significantly lower than the II values for EnB (*p* = 0.001), SB (*p* = 0.027), and SnB (*p* = 0.013) (Table 4).

## 4. Discussion

The innovative design of this study, which investigated cereal-based food products from starch structure to in vivo metabolic fate, demonstrates how food processing intrinsically modifies starch organization in cereal products, leading to different postprandial metabolic responses. We showed that rotary molding better preserves starch in its native state and slows its digestion, which led to a significantly lower GI and II compared to the other three processing technologies studied. Each food product was examined in detail using very precise imaging techniques to characterize the starch in each product and better understand the different metabolic effects of the seven cereal products. As described previously, starch granules undergo drastic structural changes during processing, with a shift from a semi-crystalline structure to an ever more amorphous one [45,46]. The imaging techniques used in this study provide new, complementary descriptions of starch within the food products: microscopy imaging provided surface information; tomography analysis generated a layer-by-layer view of the products; and crystallography provided information on the structural properties of the starch. Using these three approaches enabled us to measure starch crystallinity and assess the appearance of the starch within the matrix of each product. The X-ray diffraction patterns of the rotary-molded products revealed minimal changes in starch structure, as the diffraction pattern of the starch contained within both products (Rb and RnB) closely matched that of a typical A-type starch profile [47,48]. These observations were further highlighted by the scanning electron microscopy images, which showed clear, well-defined, and visibly intact wheat starch granules very similar to those found in raw wheat flour [49]. The structural integrity of these granules was retained throughout the matrix, as determined by micro-tomography analysis. The lack of significant changes in starch configuration seems to be the main reason for the high SDS and low GI of both rotary-molded products, as SDS is primarily linked to the degree of gelatinization and directly influences the GI of a product [21]. The starch in the soft-baked cakes (SB and SnB) as well as the rusk (Rk) had undergone changes during processing. However, the starch in these products still retained part of its original structure, as shown by the rounded peaks in the diffraction patterns and the altered shapes of the starch granules visible by scanning electron microscopy and micro-tomography. One of the major differences between these three technologies, namely rotary-molded, soft-baked, and rusk (Bread Substitute), is the water content added in the dough. For the rotary molding, the very low water quantity limited starch gelatinization phenomenon during cooking, whereas, the water content was high enough for starch gelatinization in the two other food processing, as it was associated with longer cooking time [11]. The extrusion process dramatically modified the starch structure mainly due to the high pressure which disrupted the starch granule morphology inducing starch gelatinization, as detailed recently [50].

The addition of wheat bran (4% to 7%) did not modify the impact of processing on in vitro starch digestibility and starch crystallinity. Indeed, the products generated by all three food processing technologies (rotary molding, soft baking, and extrusion) exhibited similar results for all studied parameters, whether or not they contained bran. There is some evidence that the addition of wheat bran to a cereal product can decrease the glycemic response in vivo, especially for extruded products, but this difference is not statistically significant [51,52]. Adding wheat bran resulted in the products containing approximately 2 g more fiber per portion. These results confirm the usefulness of using wheat bran, or even whole grain flour, in cereal product recipes, and of selecting specific food processing technologies to lower the acute postprandial glycemic response and potentially benefit long-term health [32,53,54].

In addition to measuring glycemic parameters (GI, AUCi, and Cmax), plasma insulin was also measured to better understand how ingestion of these products modified physiological factors [22,29,36]. None of the seven products tested exacerbated insulin secretion compared to the glycemic response. Both rotary-molded biscuits provoked the lowest insulin demand compared to the other products. Lower glycemic responses associated with lower insulin demands have been linked to the prevention of metabolic diseases [36,55].

While considerable care was taken to limit differences in the ingredients and macronutrient contents of the seven cereal products, the recipes did vary somewhat. Glycerin and eggs were added to the soft-baked cake recipes to facilitate processing. These ingredients did not have a significant effect on the final macronutrient composition of the test portions. There were only minimal differences among the portions used for the in vivo study, which are unlikely to have affected the GI [56]. Indeed, an earlier study showed that a 2-g difference in protein and fat content did not influence GI [23]. While all the products contained 50 g of available carbohydrates, this comprised different proportions of starch and sugar, especially in the soft-baked cakes, which had a higher sugar content compared with the other food products (17 and 20 g/portion compared to 14–16 g/portion for the other five food products). However, when compared to other products containing the same amount of SDS, the GI values were in the same intermediate range, with no statistical difference. This is consistent with previous results showing that sugar is not one of the main factors affecting the GI of cereal-based foods [23]. The greatest difference among the food products was their SDS content, which is likely the main factor that affected the differences in their GI values, especially between the intermediate and low GI values. The differences in SDS content can be partly explained by the amount of starch that was gelatinized, as this has been shown to directly influence the rate of digestion [21,57,58].

The two cereal products with intermediate GI values (SB and Rk) and EB, which had a significantly higher GI, had similarly low SDS values (<2 g/100 g). This discrepancy could potentially be explained by the nature of the structural changes that occurred in the starch during processing. The diffraction patterns of SB and Rk were close to the typical A-type crystallinity of wheat starch, but the diffraction peaks at a 2-Theta value of 19.5° were more rounded, indicating the presence of amorphous material [11,42], as well as V-type amylose that may correspond to the formation of amylo-lipid complexes [59,60]. These complexes are usually linked to slower digestion rates, which could explain the lower GI values [61]. Thus, the amylo-lipid complexes, which may form during processing by some technologies, may have a greater influence on GI values than anticipated based on previous works, and could be used as an additional way of reducing GI [5]. In contrast, the starch structure was most heavily altered in EB compared to the other products tested. The diffraction patterns exhibited multiple new crystallinity peaks, none of which corresponded to the peaks associated with the A-Type crystallinity of wheat starch. The micro-tomography and microscopy images showed that the starch structure had been completely altered, forming a homogenous matrix that incorporated large air bubbles.

## 5. Conclusions

In summary, the results from this study linked the effect of food process on starch organization inside the food matrix, starch digestibility profile, and finally their metabolic effect. Some food processing technologies led to greater starch alteration which induced high glycemic and insulin excursions in postprandial period. By contrast, the preservation of starch structure as close as possible to its native form, i.e., intact starch granules with low porosity, as obtained when using a rotary molding approach, led to a lower glycemic response and insulin demand. Modulation of postprandial glycemia is a meaningful target in the prevention of metabolic diseases. This can be achieved through the modification of dietary factors such as starch digestibility.

## Figures and Tables

**Table 1:** Details of the ingredients of and food processing technologies applied to the seven cereal products.

Technology	Ingredients	Mixing and Forming Steps	Cooking
Rotary-molded	With Bran (RB): Wheat flour 55%, wheat bran 6%	All of the ingredients were combined, and the resulting dough was passed through a rotary molder to form individual 15-g raw biscuits.	Convection tunnel oven for 9 min (150–190 °C).
	Without Bran (RnB): Wheat flour 61%, wheat bran 0%		
	Common ingredients: Sugar 18%, rapeseed oil 12%, water 7%, salt 0.3%		
Soft-baked	With Bran (SB): Wheat flour 39%, wheat bran 4%	All of the ingredients were combined, and then 34 g of the resulting batter was piped into individual molds using a pastry bag.	Static convection oven for 15 min at 170 °C.
	Without Bran (SnB): Wheat flour 43%, wheat bran 0%		
	Common ingredients: Sugar 17%, rapeseed oil 9%, water 12%, whole eggs 12%, glycerin 6%, salt 0.2%		
Extrusion	With Bran (EB): Wheat flour 59%, wheat bran 7%	The flour and sugar were added to the extruder (BC45) (along with the bran for the wholegrain version). A filling made from sugar and fat was pumped into the formed product as it exited the extruder. The resulting products were shaped into bite-size portions using a crimper.	Static oven at 180 °C for 1.5 min to dry the products.
	Without Bran (EnB): Wheat flour 66%, wheat bran 0%		
	Common ingredients: Sugar 20%, rapeseed oil 14%, salt 0.3%		
Rusk (Bread Substitute) (Rk)	Wheat flour 44%, sugar 19%, rapeseed oil 9%, water 21%, salt 0.3%, yeast 6%	All of the ingredients were combined, and the dough was kneaded for 15 min using a mechanical kneader. Because of its consistency, 400-g portions of dough were transferred to individual molds. The dough was then left to proof for 15 min.	Static convection oven for 40 min (150 to 200 °C). Once out of the oven, the loaves were allowed to cool overnight in a fridge set at 8 °C. They were then sliced and toasted at 160 °C for 13 min to obtain the final rusk products.

**Table 2:** Product portion size and macronutrient content per portion.

Nutritional Composition of Products	Reference Food Glucose	Rotary- Molded Biscuit with Bran RB	Rotary- Molded Biscuit without Bran RnB	Soft-Baked Cake with Bran SB	Soft-Baked Cake without Bran SnB	Rusk Rk	Extruded Product with Bran EB	Extruded Product without Bran EnB
Portion size (g)	51-g glucose solution	69	66	93	89	69	75	72
Moisture (g)	-	0.5	0.9	16.4	12.5	1.1	4.4	4.4
Protein (g)	0.0	4.6	4.2	6.1	5.2	5.7	4.8	4.4
Fat (g)	0.0	9.6	10.9	11.7	12.0	10.0	10.9	10.1
Sugar (g)	50.0	15.2	15.9	19.7	17.0	16.5	15.4	13.9
Available Starch (g)	0.0	31.7	29.7	28.8	28.4	30.7	30.9	33.3
Available carbohydrate (g)	50.0	50.0	50.0	50.0	50.0	50.0	50.0	50.0
Fiber (g)	0.0	4.0	1.4	4.2	1.9	1.9	4.1	1.8
Calculated energy (kJ)	800	1265	1286	1369	1348	1283	1319	1261

**Table 3:** Starch digestibility fractions in each product.

Starch Fractions in Products	Rotary-Molded Biscuit with Bran RB	Rotary-Molded Biscuit without Bran RnB	Soft-Baked Cakes with Bran SB	Soft-Baked Cakes without Bran SnB	Rusk Rk	Extruded Product with Bran EB	Extruded Product without Bran EnB
SDS (g/100 g)	23.9	27.5	1.5	1.5	1.1	0.1	0.1
RDS (g/100 g)	26.7	24.1	32.4	35.7	47.4	46.0	53.1
RS (g/100 g)	0.5	0.4	0.3	0.4	0.6	0.4	0.7

SDS, slowly digestible starch; RDS, rapidly digestible starch; RS, resistant starch.

**Table 4:** Subjects’ postprandial metabolic responses parameters (*n* = 12).

Blood Glucose and Insulin Parameters	Reference Food Glucose	Rotary-Molded Biscuit with Bran RB	Rotary-Molded Biscuit without Bran RnB	Soft-Baked Cakes with Bran SB	Soft-Baked Cakes without Bran SnB	Rusk Rk	Extruded Product with Bran EB	Extruded Product without Bran EnB
Glucose parameters								
Baseline blood glucose level (mmol/l)	5.1 ± 0.1	5.1 ± 0.1	5.1 ± 0.1	5.1 ± 0.1	5.0 ± 0.1	5.0 ± 0.1	5.1 ± 0.1	5.1 ± 0.1
Cmax(g) (mmol/l))	7.5 ± 0.2 ^a^	6.0 ± 0.1 ^cd^	5.8 ± 0.1 ^d^	6.2 ± 0.2 ^b^	6.1 ± 0.2 ^ab^	6.5 ± 0.2 ^ab^	6.4 ± 0.2 ^b^	6.2 ± 0.2 ^bc^
iAUC(g) (mmol × min/l)	186 ± 13 ^a^	91 ± 16 ^cd^	79 ± 8 ^d^	117 ± 8 ^bc^	142 ± 13 ^ab^	113 ± 14 ^ab^	124 ± 14 ^bc^	114 ± 12 ^bcd^
GI (%)	100 ± 0	47 ± 5 ^d^	43 ± 3 ^d^	66 ± 4 ^abc^	77 ± 4 ^a^	63 ± 6 ^bc^	66 ± 5 ^abc^	61 ± 4 ^bc^
Insulin parameters								
Baseline insulin level (pmol/l)	33.7 ± 1.7	35.5 ± 2.3	37.3 ± 2.1	37.4 ± 2.8	35.6 ± 2.5	36.8 ± 2.1	36.2 ± 2.2	35.5 ± 1.5
Cmax(ins) (pmol/l)	383 ± 25 ^a^	240 ± 24 ^cd^	231 ± 21 ^d^	312 ± 28 ^abcd^	338 ± 37 ^ab^	325 ± 30 ^abc^	326 ± 34 ^ab^	299 ± 24 ^bcd^
iAUC(ins) (pmol × min/l)	2525 ± 197 ^a^	1438 ± 183 ^cd^	1350 ± 122 ^d^	2055 ± 214 ^abcd^	2252 ± 228 ^ab^	2177 ± 277 ^abc^	2073 ± 260 ^ab^	1593 ± 111 ^bcd^
II (%)	100 ± 0	56 ± 4 ^d^	54 ± 4 ^d^	76 ± 4 ^abc^	85 ± 6 ^bc^	79 ± 4 ^bc^	80 ± 6 ^bc^	65 ± 4 ^ad^

Glucose parameters: baseline, maximum concentration (Cmax(g)), incremental area under the curve (iAUC(g)), and glycemic index (GI). Insulin parameters: baseline, maximum concentration (Cmax(ins)), incremental area under the curve (iAUC(ins)), and insulin index (II). Statistical differences (*p* < 0.05) are represented by different letters. Mean ± SEM.
